# Non-Criteria Obstetric Antiphospholipid Syndrome: Myth or Reality?

**DOI:** 10.3390/jcm14041299

**Published:** 2025-02-15

**Authors:** Sara Beça, Maria Borrell, Ricard Cervera, Francesc Figueras, Alfons Nadal, Gerard Espinosa, Núria Baños

**Affiliations:** 1 Department of Autoimmune Diseases, Reference Centre for Systemic Autoimmune Diseases (UEC/CSUR) of the Catalan and Spanish Health Systems-Member of ERNReCONNET, Hospital Clínic, University of Barcelona, 08036 Barcelona, Spain; sidossantos@clinic.cat (S.B.); rcervera@clinic.cat (R.C.); 2 Department of Maternal-Fetal Medicine, BCNatal, Barcelona Centre for Maternal-Fetal and Neonatal Medicine, Hospital Sant Joan de Déu and Hospital Clínic, University of Barcelona, 08028 Barcelona, Spain; mborrellb@clinic.cat (M.B.); ffiguera@clinic.cat (F.F.); nbanos@clinic.cat (N.B.); 3 Department of Pathology, Hospital Clínic, University of Barcelona, 08036 Barcelona, Spain; anadal@clinic.cat

**Keywords:** non-criteria obstetric antiphospholipid syndrome, low-titer aPL, infertility, recurrent implantation failure

## Abstract

Women with adverse pregnancy outcomes suggestive of obstetric antiphospholipid syndrome (OAPS), but not fulfilling clinical and/or laboratory international classification criteria, are increasingly recognized both in clinical practice and in the literature. This entity is termed non-criteria OAPS (NC-OAPS). It includes clinical scenarios such as two unexplained pregnancy losses, three non-consecutive pregnancy losses, late pre-eclampsia/eclampsia/signs of placental insufficiency, or recurrent implantation failure, as well as positive low-titers of antiphospholipid antibodies (aPLs) and non-classical aPLs. To address the NC-OAPS heterogeneity, a nomenclature proposal was developed. In recent years, retrospective and prospective cohort studies have been designed to clarify the characteristics and outcomes of the different subsets of NC-OAPS. In general, the studies support that NC-OAPS may benefit from treatment with antithrombotic, anticoagulant and/or immunomodulator agents, but several considerations must be made on the robustness and nuances of the scientific evidence. The objective of this review is to critically analyze the available evidence supporting the diagnosis of NC-OAPS, categorize its subsets, and evaluate the impact of treatment strategies on its outcome. We also remark on questions that are still unanswered, such as the lack of consensus on diagnostic criteria or treatment protocols.

## 1. Introduction

Antiphospholipid syndrome (APS) is a systemic autoimmune disorder characterized by vascular thrombosis and/or pregnancy morbidity in the presence of antiphospholipid antibodies (aPLs) [1]. More recently, aPL-associated “non-criteria” non-thrombotic manifestations (such as livedo racemosa, cardiac valve thickening, cognitive impairment, and thrombocytopenia) have been recognized and aggregated to the spectrum of the syndrome [2]. Obstetric antiphospholipid syndrome (OAPS) is a subset of APS characterized by persistently positive aPLs and obstetrical complications, which include early pregnancy losses, late fetal losses, and preterm delivery < 34 weeks of gestation because of severe pre-eclampsia (PE)/eclampsia or placental insufficiency (PI) [1].

APS classification criteria have been created to define homogeneous and well-selected cohorts appropriate for clinical research. The first APS classification criteria were formulated in Sapporo in 1999. These original classification criteria included only moderate–high titers (i.e., >40 GPL or MPL, or >the 99th percentile) of anti-cardiolipin (aCL) IgG/IgM and lupus anticoagulant (LA) as laboratory criteria [3]. In 2006, a revised version of the Sapporo criteria was designed at a consensus workshop in Sidney. In these criteria, the anti-β2 glycoprotein-I antibody (aβ2GPI) IgG/IgM antibody was included (in titer > the 99th percentile), and the requirement of the persistent presence of aPLs in ≥two occasions at least 12 weeks apart was added [1]. The more recent American College of Rheumatology (ACR)/European Alliance of Associations for Rheumatology (EULAR) APS classification criteria, published in 2023, include a scoring system in which the criteria are differently weighted and organized into six clinical and two laboratory domains [2]. To be classified as APS, patients should accumulate at least 3 points each from the clinical and laboratory domains. Also, in these new criteria, the presence of only aCL or aβ2GPI IgM isotypes without IgG positivity is attributed a lower weight (1 point). Regarding the obstetrical complications, the various criteria include, in general, the same type of manifestations: ≥three early pregnancy losses, at least one fetal loss >10 weeks of gestation of a morphologically normal fetus or preterm delivery < 34 weeks gestation because of severe PE/eclampsia or placental insufficiency (PI) [2]. However, the 2023 criteria introduce more stringent details for pregnancy morbidity, attributing a higher weight to the presence of severe PE/eclampsia and to PI. In the absence of PE/eclampsia/PI, all other obstetric manifestations are only attributed 1 point (Table 1) [2]. In these new classification criteria, there is a decrease in their sensitivity linked to the obstetric domain, with early and late fetal loss underrepresented, as well as patients carrying only IgM aCL/aβ2GPI antibodies [4,5]. Until this day, most of the available and recent research cohorts are classified according to the Sydney criteria and this review will reflect that view.

Classification criteria intentionally include very stringent definitions to accomplish very high specificity. For this reason, their strict application in routine practice to diagnose individual patients should be avoided, and more emphasis should be given to the broad range of APS features, to the existing complementary tests in any given scenario and in the exclusion of alternative diagnoses. In daily clinical practice, specifically in the obstetric field and more so with the new ACR/EULAR criteria, physicians are frequently faced with patients with clinical features and laboratory manifestations suggestive of OAPS but who do not strictly meet the classification criteria. These patients are globally described as having non-criteria obstetric antiphospholipid syndrome (NC-OAPS) [3]. Evidence is accumulating on the potential clinical significance of NC-OAPS, suggesting that its diagnosis and treatment may have impact on maternal and fetal outcomes. However, there is much heterogeneity between the scenarios and no consensus on the features to include in the definition of NC-OAPS. Consequently, determining the optimal treatment is challenging. Currently, the treatment of NC-OAPS requires an individualized strategy according to the patient’s risk profile, although the risk factors in NC-OAPS are not completely explored.

In this review, we examine the available evidence supporting the diagnosis of NC-OAPS, the clinical relevance of its different subsets, and the impact of treatment strategies.

## 2. Definition and Diagnosis of NC-OAPS

The first limitation for the diagnosis of NC-OAPS resides on the diversity of the definitions used in the literature. Based on the different scenarios seen in clinical practice and depicted in the literature, a nomenclature proposal for “non-criteria” APS subtypes was suggested by our group [6]. According to this proposal, four APS patient profiles are recognized: (a) patients with clinical APS criteria, plus the presence of “non-criteria” manifestations but persistently negative aPLs (seronegative APS); (b) patients with “non-criteria” manifestations plus aPL positivity fulfilling the APS laboratory criteria (clinical non-criteria APS); (c) patients with clinical APS criteria, plus positive aPLs, but not fulfilling the laboratory classification criteria because of persistently positive but low-titer (between the 95th and 99th percentiles or below 40 GPL or MPL) aPLs (incomplete laboratory APS); and (d) patients with APS clinical criteria manifestations (thrombotic or obstetric), with persistently negative or low-titer criteria aPLs, but positive aPLs different from LA/aCL/aβ2GPI IgM or IgG isotypes (laboratory non-criteria APS) (Table 2) [6]. There are other groups of patients frequently present in the literature that were excluded from this nomenclature proposal, although they can have relevance in the spectrum of “non-criteria” APS. These groups include aPL carriers, patients with “non-criteria” manifestations and positivity for “non-criteria” aPLs, and patients with clinical manifestations fulfilling APS classification criteria and only one single positive determination of criteria aPLs (referred as single-positive APS).

According to this nomenclature proposal, from a laboratory point of view, the diagnosis of NC-OAPS may be considered in patients with low titers of aPLs or with non-classical (non-criteria) aPLs (autoantibodies directed against phospholipids, phospholipid-binding proteins or protein–phospholipid complexes apart from the conventional criteria antibodies LAs, aCLs and aβ2GPIs). From a clinical perspective, several pregnancy complications not included within the Sydney criteria were identified. These are the “non-criteria” obstetric manifestations of APS. In the literature, the term aPL-related obstetric morbidity (OMAPS) is also used to refer to these heterogenous obstetric clinical manifestations that do not fulfill the Sydney clinical criteria. Based on the reports from the multicenter European Registry on Obstetric Antiphospholipid Antibody Syndrome (EUROAPS), the following “non-criteria” obstetric manifestations were recognized: one or two unexplained pregnancy losses before 10 weeks of gestation, three non-consecutive pregnancy losses, late placental vasculopathy (PE after 34 weeks, intrauterine growth restriction after 34 weeks), late premature birth (after 34 weeks and under 37 weeks of pregnancy) with no other apparent cause, placental abruption, placental hematoma, puerperal PE, and recurrent implantation failure (RIF) [7]. These OMAPSs are in general less numerous and/or less devastating than those used in the APS classification criteria. However, as pregnancies are limited events in the life of a woman, all the obstetric outcomes potentially related to aPLs are viewed as preventable clinical opportunities. For example, in a woman carrying aPLs and with two previous unexplained early pregnancy losses, it is reasonable to assume the possible diagnosis of NC-OAPS and offer treatment in the next pregnancy, potentially avoiding another adverse outcome. In routine clinical practice, the most frequent scenario is composed of women with low and/or fluctuant aPL titers discovered in the context of an unfavorable pregnancy event. In a woman with a large burden of obstetric complications, a future adverse pregnancy outcome (APOs) may be anticipated and offering treatment is usually acceptable. Conversely, the role of low aPL titers in a woman with only one previous pregnancy loss may be more debatable. For these reasons, it is very important to be able to diagnose NC-OAPS with accuracy, understand its nuances, and comprehend its relevance in clinical practice.

## 3. A Glimpse into Pathophysiology

It is now accepted that thrombosis alone cannot explain the pregnancy morbidity associated with aPLs. In OAPS, aPLs target the placenta, with animal models demonstrating the direct aPL-mediated functional damage on placental tissue and/or the inflammatory processes [8]. Multiple aPL-mediated actions on the different cellular components of placental tissue may act together or in various combinations at different times of pregnancy, resulting in defective placentation. The biologic mechanisms potentially implicated in the pathogenesis of aPL-associated obstetric morbidity include trophoblast dysfunction and apoptosis, decidual inflammation, the disruption of annexin A5 shield on placental villi surfaces leading to phosphatidylserine exposure, the inhibition of endometrial angiogenesis, complement activation, neutrophil infiltration, local tumor necrosis factor secretion, and the formation of neutrophil extracellular traps in the intervillous spaces [8,9].

**Figure 1:**
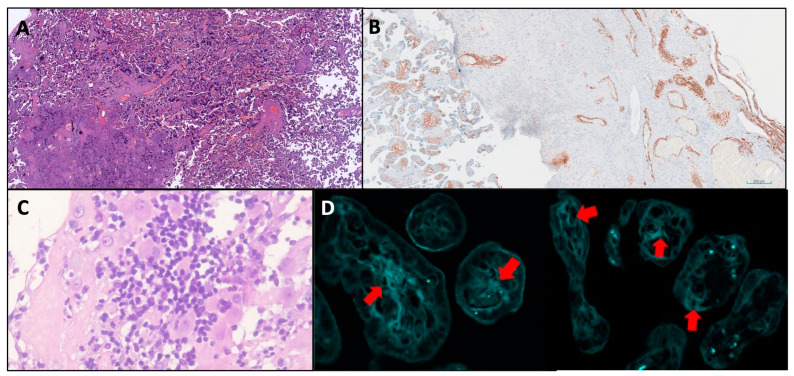
(**A**) Placental infarction (villous infarction) with intervillous space collapse and coagulative necrosis of chorionic villi in the left lower quadrant. Syncytial knot hyperplasia, visible as multiple dark hematoxylin-stained sprouts upon chorionic villi. H&E staining. Original magnification 2×. (**B**) Impaired spiral artery remodeling with persistence of the muscular wall (brown structures on the right side). Smooth-muscle actin immunohistochemistry counterstained with hematoxylin. Original magnification 4×. (**C**) Decidual inflammation. Chronic deciduitis is characterized by a dense lympho-plasmacytic inflammatory infiltrate dissecting decidual cells. H&E staining. Original magnification 40×. (**D**) The deposition of complement split product C4d signed with red arrows. Original magnification 40×.

From a placental histopathologic point of view, a systematic review found that the following features were reported more frequently in the placentae of aPL-positive women compared to aPL-negative women: placental infarction, impaired spiral artery remodeling, decidual inflammation, an increase in syncytial knots, decreased vasculosyncytial membranes and the deposition of complement split product C4d (Figure 1) [10].

Interestingly, a case–control study including 27 subjects with primary APS, 51 with NC-OAPS, 24 with aPLs associated with other connective tissue disorders (CTD), and 107 healthy controls found that placental lesions suggesting severe maternal vascular malperfusion were more common among primary (odds ratio [OR] 11.7, 95% CI 1.3–10.8) and NC-OAPS (OR 8.5, 95% CI 1.6–45.9) compared with the controls. The risk of fetal vascular malperfusion was higher in primary APS (OR 4.5, 95% CI 1.2–16.4), aPL associated with CTD (OR 3.1, 95% CI 1.5–6.7) and NC-OAPS (OR 5.9, 95% CI 1.7–20.1) compared with the controls. These observations indicate that, although the rates and severity of several types of maternal vascular malperfusion lesions are higher in primary APS, they are also observed in patients with NC-OAPS in a higher frequency than in the controls [11].

In clinical practice, the comprehensive histopathologic analysis of placentas deriving from pregnancies with adverse outcomes, searching for the described placental histopathologic features, may contribute to the diagnosis of NC-OAPS in cases where the fluctuant or low titers of aPL shadow the diagnosis. Also, according to the demonstrated role of thrombosis, inflammation and complement activation in aPL-related pregnancy morbidity, treatment rationale focuses on intervention in these areas. Indeed, inflammation at the placental level is more relevant than in vascular APS, supporting the attempts to use anti-inflammatory agents as adjuvant treatments in this condition.

## 4. The Relevance of the Different Types of Non-Criteria Obstetric Manifestations

The prevalence of aPLs, and hence the role of these antibodies, in specific cohorts of patients with APS-related obstetrical complications not meeting the Sydney criteria was addressed in few studies, and those that exist have several limitations, such as small samples of patients, the inclusion of women with criteria obstetrical manifestations, testing only part of the aPLs, or not considering all other causes of the adverse obstetric events.

Early recurrent pregnancy loss has been found as the most frequent obstetrical complication in OAPS, described between 35.4% and 38.6% of patients in large cohorts [12,13]. However, the specificity of recurrent early miscarriages is low due to the difficulty in fully excluding other potential causes. It is particularly important to consider this limitation when contemplating the diagnosis of NC-OAPS in the case of ≤three early pregnancy losses. A chromosomal abnormality is the most common cause of pregnancy losses, and an effort should be made to attain this information, although it is not always feasible. Conversely, patients with two previous early pregnancy losses, particularly when embryos are euploid, might eventually fulfill the classification criteria for OAPS if the woman embarks on a new pregnancy, especially if a diagnostic assessment or treatment is not offered in the meanwhile. This is indeed a classic example of the inevitable divergence between diagnostic and classification OAPS criteria in clinical practice.

A large trial examining the prevalence of aPLs in placenta-mediated complications (small-for-gestational-age neonate [*n* = 378], PE with severe features [*n* = 30], and placental abruption [*n* = 23]) in patients delivering after >36 gestational weeks found a prevalence of aPLs of 4.9% in the entire cohort, and this was comparable between the three subgroups (small-for-gestational-age, 3.9%; PE with severe features, 3.3%; and placental abruption, 13%) [14]. This prevalence is similar to that reported in the general obstetric population (ranging between 1.4 and 7%) [14]. A previous study exploring the prevalence of aPLs in late-onset pregnancy complications (>28 gestational weeks) in 100 patients and comparing it with healthy controls with uneventful pregnancies found a higher prevalence of aPLs (31%), resulting in a fourfold risk for any late-onset pregnancy complication. However, this study also included non-classical aPLs, as well as stillbirth and placenta-mediated complications occurring under 34 weeks of gestation (which are included in the APS criteria), and these differences could explain the higher aPL prevalence found [15].

The causal link between aPLs with infertility or RIF in those undergoing in vitro fertilization (IVF) is a matter of debate. A systematic review estimated positivity rates of criteria and non-criteria aPL tests of 6% and 3% among infertile women and of 1% and 2% among control subjects, respectively [16]. A significant difference in the positivity rate of patients versus controls emerged for aCLs only. However, this study highlighted several limitations of the available studies, including wide heterogeneity in study populations, aPL cutoffs not conforming to international guidelines in more than 75% of studies, aPL positivity not confirmed in 89% of studies, and methodological issues such as inappropriate study design. These limitations precluded more robust statistical analyses and conclusions [16]. The data regarding the association of aPLs with IVF outcomes are also conflicting. A meta-analysis of only prospective studies addressing the IVF outcomes in infertile patients found no significant association between aPL positivity and IVF outcomes, namely clinical pregnancy, miscarriage and live births [17]. However, two other meta-analyses concluded that patients with RIF or at least two implantation failures had a higher prevalence of aPLs than women with a successful implantation or healthy fertile women [18,19]. The strength of association varied according to different aPL subtypes and between the studies and did not prove causality.

Globally, the studies addressing the impact of aPLs on obstetric events different from the APS criteria manifestations suffer from a lack of adequate study populations and standardized research methodologies (small or heterogenous samples, an incomplete consideration of other causes of adverse pregnancy outcomes that may act as confounders, and large variations in aPL detecting methods and cutoffs). These limitations preclude definite conclusions regarding the causal relationship between aPLs and these events and, as a consequence, cause uncertainty on how to manage them.

## 5. The Relevance of the Non-Criteria Laboratory Manifestations

The titers of aPLs and their clinical significance have many particularities. For one, it is recognized that aPL levels can fluctuate over time, even becoming undetectable, but evidence on the outcomes of patients according to the degree of fluctuation is sparse [20]. The aPL level that is associated with a clinical risk has not been established in practice, but more than 10 years ago, authors started to report obstetrical complications in patients with low aPL levels [21]. One limitation that should be taken into account is the matter of agreement between the test results of different laboratories, which has been shown to be inferior in lower positive titers than in moderate-to-high titers. Also, it is recognized that testing for APS can be affected by pregnancy itself, and it should be performed between pregnancies wherever possible [20].

Studies have shown that low levels of IgG/IgM aCL levels and/or IgM aβ2GPI are more frequent in patients with only obstetric morbidity compared to those with thrombosis [22,23]. Other studies reported that the probability of obstetric events without treatment was also similar in women with low titers and women with high titers of aPLs and higher than in aPL-negative controls [24,25,26]. These observations support the concept of incomplete laboratory APS as a subset of NC-OAPS.

Patients with only one positive aPL determination (single positivity) are challenging because it is difficult to exclude a false positive aPL elevation due to other causes (e.g., infection, malignancy, or drugs), and previous data have indicated that these patients have no increased risk of recurrent events compared to those without aPLs [6]. However, it was also noted that a single-positive aPL test is more common in purely obstetrical APS than in thrombotic or mixed APS [27]. Therefore, the relevance of a single positive result of aPLs remains inconclusive.

In patients with clinical criteria for OAPS but seronegative for conventional aPLs, considerable efforts have been undertaken in recent decades to discover new aPLs with clinical relevance. Various antibodies are promising, such as IgA aβ2GPI and aCLs and those against annexin A5, prothrombin, phosphatidylserine/prothrombin (aPS/PT), phosphatidylethanolamine, phosphatidic acid, phosphatidylserine, phosphatidylinositol, vimentin/cardiolipin complex, β2GPI Domain 1, and β2GPI/HLA-DR complex [6,28,29]. Many patients with seronegative APS could have one or more of these antibodies if they were available in routine clinical practice. Non-conventional aPLs were reported in 68% of these patients [30]. In a large Chinese cohort composed of 192 APS patients, 90 seronegative APS patients, 193 autoimmune disease controls, and 120 healthy controls, ten aPLs were tested, including five non-criteria aPLs: aPS/PT IgG/IgM, aCL IgA, aβ2GPI IgA, and anti-β2GPI Domain IgG. At least one non-criteria aPL was detected in 60.9% of seronegative APS and 93.5% of APS patients [28]. The aPS/PT IgG and aPS/PT IgM antibodies were the most frequently detected aPLs in both APS and seronegative APS patients. The aPS/PT antibodies are indeed amongst the best studied non-criteria aPLs. There are studies with experimental models that describe their pathogenic role inducing thrombosis [31,32], and many cohort studies with multivariate analysis support that aPS/PT antibodies are independent risk factors for obstetric complications, although not all studies reach the same conclusion, and controversy exists around the impact of each isotype and on different types of obstetric complications [33]. A limitation to the use of non-criteria antibodies is the lack of the validation of the laboratory assays and respective titers.

In summary, the current evidence supports the value of low levels of aPLs in a compatible clinical scenario. In patients with a high clinical suspicion of OAPS but with negative aPLs, it should be remembered that other pathogenic antibodies not detected by the usually available laboratory assays may be playing a role. In these cases, the search for non-classical aPLs may be considered if the testing is accessible (particularly in a research setting) while being knowledgeable of its limitations (the lack of validation and standardization).

## 6. Comparison Between NC-OAPS and OAPS

There are two large comparative studies of the features and pregnancy outcomes in OAPS and NC-OAPS. In the EUROAPS study, a large-scale international multicenter registry analysis, patients with OAPS had a higher number of previous miscarriages, fetal losses, stillbirths, early placental vasculopathies (PE < 34 weeks and FGR < 34 weeks) and prematurity than those included in the NC-OAPS group. Women diagnosed with NC-OAPS had higher rates of RIF and late placental events (PE > 34 weeks and FGR > 34 weeks) [7]. These observations are, at least in part, the result of the definitions of OAPS and NC-OAPS. Interestingly, the authors of the study also found differences in the outcomes between the two groups. Even though the rate of live births was similar, obstetric complications occurred in 73.4% of pregnancies in NC-OAPS and in 65.1% of pregnancies in OAPS (*p* < 0.001) [7]. In a large Chinese series including 1006 patients (OAPS n = 141, and NC-OAPS n = 865), the previous history of >three spontaneous pregnancy losses was the only significant difference between OAPS and NC-OAPS in terms of previous clinical manifestations (19.9% in OAPS vs. 8.3% in NC-OAPS, *p* < 0.001) [34]. Regarding pregnancy outcomes, this study obtained different data to that from the EUROAPS. The authors observed that only the rate of stillbirth was statistically different between groups, being higher in the OAPS group (8.5% vs. 2.0%, *p* < 0.001). Importantly, the study did not include early pregnancy losses as an adverse outcome. After logistic regression analysis, the study found a higher stillbirth risk (OR 2.731, CI 95% 1.104–6.754) but a lower preterm risk (OR 0.486, CI 95% 0.246–0.959) in the OAPS group compared to the NC-OAPS group, but the NC-OAPS group has a significantly higher overall risk of APOs (OR 0.565, CI 95% 0.336–0.950). Double aPL positivity, triple aPL positivity (a combination of positive LA, positive aCL [IgG and/or IgM], and positive aβ2GPI [IgG and/or IgM]) and gestational hypertension were independently associated with higher odds of APOs in the OAPS group, whereas two of the double aPL positivity subtypes, triple aPL positivity and placenta previa were independent risk factors related to APOs in the NC-OAPS group [34]. Despite the differences in the results of the mentioned studies, both highlight that NC-OAPS in its general concept may be associated with APOs.

From another perspective, in a study of 163 patients (62 subjects with complete APS [38%], 48 with NC-APS [29.4%] and 53 [32.5%] asymptomatic aPL carriers) and 785 healthy controls, non-criteria and asymptomatic subjects had increased risks of a spontaneous abortion, FGR, PE and overall APOs compared to negative controls, although the size of the effect was significantly lower than that associated with complete APS [35]. The rates of APOs were 5.6% in controls, 41.9% (OR = 6, 95% CI = 2.7–13.5) in APS, 25% (OR = 4.4, 95% CI = 2–9.4) in non-criteria and 28.3% (OR = 4, 95% CI = 1.8–8.8) in aPL carriers [35].

While studies show different results in some clinical outcomes between OAPS and NC-OAPS, their interpretation should be approached with caution, as the definition of these outcomes varies between studies. Despite this limitation, it appears that NC-OAPS may behave differently from OAPS, although adverse outcomes may be expected in both.

## 7. To Treat or Not to Treat NC-OAPS?

### 7.1. Current Treatment Guidelines and Recommendations

The 2019 EULAR guidelines recommend the combination of low-molecular-weight heparin (LMWH) at prophylactic doses and low-dose aspirin (LDA) during pregnancy in women with pure OAPS who have a history of ≥three early pregnancy losses (<10-week gestation) or ≥1 late loss (≥10-week gestation) [36]. This recommendation is supported by a more recent Cochrane review (2020) that included 11 studies (1672 women) and evaluated the efficacy of aspirin or heparin or both to reduce pregnancy complications in women with APS, finding that heparin plus aspirin increased the number of live births in women with APS compared to aspirin alone (RR 1.27, 95% CI 1.09–1.49, 5 studies, 1295 women, low-certainty evidence) [37]. For patients with a history of delivery < 34 weeks of gestation due to eclampsia or severe PE or due to features of PI, the EULAR guidelines propose both treatment with LDA or LDA plus heparin at prophylactic dosage considering the individual’s risk profile. This recommendation is based on the lack of evidence that, in these patients, treatment with LDA plus heparin improves the likelihood of live births compared to treatment with LDA alone [36,38].

In the case of NC-OAPS, there is no consensus on management. The 2019 EULAR guidelines suggest considering the use of either LDA alone or in combination with LMWH, mainly based on expert opinion [36]. In the 2020 ACR guidelines, only LDA is recommended as PE prophylaxis, and there is a conditional recommendation against using a combination of prophylactic-dose heparin and LDA for patients with positive aPLs who do not meet the criteria for OAPS [39].

### 7.2. Comparative Outcomes of Treatment in OAPS and NC-OAPS

A systematic review compared the treatment and outcomes of patients with OAPS and NC-OAPS. In nine studies comparing treatment frequency in obstetric patients, eight (2762 participants) described similar treatment frequencies, and only one reported more frequent treatment in definite OAPS (179 participants) [40]. In the ten studies reporting a statistical comparison of the pregnancy outcomes of NC-OAPS versus APS, seven found similar outcomes in terms of successful pregnancies/live births (1171 participants), and the remaining three (1830 participants) even described worse outcomes/increased complications at least in some subsets of NC-OAPS in comparison with definite APS. Additionally, five studies described an improvement in live births in both NC-OAPS and OAPS with treatment [40]. The review only included cohort studies, with heterogeneous study populations, design, and treatment regimens, with a global moderate quality. There are no case–control studies or randomized trials in this area. Table 3 summarizes the characteristics and main results of the most relevant studies comparing the outcome of NC-OAPS with OAPS. In this setting, the two large cohorts previously described in this review are worth another reference. In the European registry EUROAPS, the largest study included in the mentioned systematic review, the percentage of patients treated was similar in the OAPS and NC-OAPS groups, with the observed rate of live births similar in both groups (72.8% in OAPS and 73.43% in NC-OAPS), although obstetric complications occurred in 651/1000 (65.1%) in OAPS and in 470/640 (73.4%) in NC-OAPS (*p* < 0.001) [7]. Prematurity was the most frequent complication observed in the OAPS group (24.1%) and miscarriage was the most frequent in NC-OAPS (19.4%) [7]. The other study was a retrospective single-center study with a Chinese cohort comprising 1006 patients, published after the systematic review [34]. In this study, all patients had some form of treatment, although a smaller proportion of women with OAPS received LDA alone compared with the NC-OAPS group, and a higher proportion of patients received LDA combined with LWMH plus hydroxychloroquine (HCQ) or glucocorticoids [34]. The OAPS patients had a significantly higher risk for stillbirths compared to the NC-OAPS patients, while the NC-OAPS group had a significantly higher risk for preterm birth and overall APOs [34].

The diversity of the study populations in these publications refers not only to the various NC-OAPS subtypes included but also to the inclusion of conditions that may act as confounders, for example, systemic autoimmune diseases and hereditary thrombophilia. The association between hereditary thrombophilia and APOs is controversial [41,42,43], and it is seldom evaluated or referred to in published studies. From all the studies depicted in Table 3, only three described the prevalence of inherited thrombophilia in the women included [7,21,44]. All three found that the prevalence of inherited thrombophilia was similar in OAPS and NC-OAPS: in Mekinian A et al., 0% in both groups; in Alijotas-Reig J et al., 15.9% in the OAPS versus 17.3% in the NC-OAPS group; and in Martinez-Taboada VM et al., 18.5% in OAPS versus 13.0% in the NC-OAPS group. The thrombophilia types are not specified.

In summary, most (but not all) studies reveal that, in pregnancies of patients with NC-OAPS, treatment is used in a similar frequency than in OAPS, achieving a comparable and improved live birth rate. The comparative risk associated with both global and specific types of APOs is less well defined.

**Table 3:** Main studies comparing outcomes of pregnancies in women with OAPS and NC-OAPS.

Authors (Ref)	Year	Design and Setting	Study Population	Number of Patients	Objective	Main Results
Liu H et al. [34]	2024	Retrospective Single-Center	Includes the following: (1) OAPS (2) NC-OAPS (aPL-related pregnancy morbidity [two unexplained miscarriages, ≥three non-consecutive miscarriages, late PE, placental abruption, late premature birth, and two or more unexplained IVF failure), low-titer and/or non-persistent aPL) Excludes other SADs No inclusion of non-classical aPL	OAPS: 141 NC-OAPS: 865	- To compare clinical characteristics and obstetric outcomes between primary OAPS and NC-OAPS - To explore the risk factors for APOs in both groups	- OAPS patients had a significantly higher risk for stillbirths compared to the NC-OAPS patients, while the NC-OAPS group had a significantly higher risk for preterm birth and overall APOs. - Double aPL positivity, triple aPL positivity, and gestational hypertension were the independent risk factors for APOs in OAPS patients. - Two of the double aPL positivity subtypes, triple aPL positivity and placenta previa were independent risk factors for APOs in NC-OAPS patients.
Chen J et al. [45]	2024	Retrospective Single-Center	Includes the following: Patients with ≥2 pregnancy losses with (1) Medium-high-titer aPL (2) Low-titer APL Excludes other SADs No inclusion of non-classical aPL	Medium–high-titer aPL: 32 Low-titer-aPL: 92	- To investigate the impact of low-titer aPLs in patients with recurrent pregnancy loss - To compare pregnancy outcomes between patients with low and medium–high aPL levels.	- Appropriately treated patients in both low-titer and medium–high-titer aPL positivity achieved higher live birth rates (33.3% vs. 67.6% in low titers and 66.7% vs. 79.3% in the medium–high-titer group).
Martínez-Taboada, VM et al. [44]	2022	Retrospective Single-Center	Includes the following: (1) OAPS (2) NC-OAPS (aPL-related pregnancy morbidity, low-titer or intermittent positive aPL) (3) Seronegative APS group Excludes other SADs No inclusion of non-classical aPL	OAPS: 66 NC-OAPS: 140 Seronegative APS: 57	- To compare characteristics and fetal–maternal outcomes between women with OAPS, NC-OAPS and seronegative APS	- Patients with OAPS received more intensive treatment with LMWH+LDA (75.8% in OAPS and 45.7% in NC-OAPS patients). - The live birth rate was similar between groups, with and without treatment, but APOs are more frequent in OAPS after treatment. - SoC treatment increased the live birth rates in both groups (75.4% in OAPS and 70.7% in NC-OAPS).
Spinillo et al. [35]	2021	Prospective Single-Center	Includes the following: (1) APS (2) NC-APS (aPL-related pregnancy morbidity, low-titer or intermittent positive aPL) (3) aPL carriers (asymptomatic) (4) Control group (healthy pregnant women) Includes patients with SAD and thrombotic APS No inclusion of non-classical aPL	APS: 62 NC-APS: 48 aPL carriers: 53 Control: 785	- Evaluate the rate of obstetric complications and the burden of obstetric outcomes in APS, NC-APS and asymptomatic aPL carriers	- All the categories of women with aPLs have an increased incidence of APOs. - LMWH plus LDA was carried out in 85.5% of subjects with complete APS, 4.2% of NC-APS and none of aPL carriers. - The rate of APOs was 5.6% in controls, 41.9% (adj.OR = 6, 95%CI = 2.7–13.5) in APS, 25% (adj.OR = 4.4, 95%CI = 2–9.4) in NC-APS and 28.3% (OR = 4, 95%CI = 1.8–8.8) in aPL carriers. - SADs were independently associated with an increased risk of adverse obstetric outcomes
Li X et al. [46]	2021	Prospective Single-Center	Includes the following: (1) OAPS group (2) NC-OAPS group (aPL-related pregnancy morbidity, low-titer and/or non-persistent aPL) Includes patients with SAD No inclusion of non-classical aPL positivity	APS: 34 NC-OAPS: 94	- To assess possible factors related to the pregnancy outcomes of patients with positive aPL and APO histories - To compare criteria OAPS and NC-OAPS patients	- Similar live births in OAPS and NC-OAPS (76.5% and 74.5%, respectively) when treated. - Pre-eclampsia or eclampsia before the 37th gestational week was significantly higher in the OAPS group. - In NC-OAPS, LMWH was a protective factor for APOs. The percentage of APOs in the LDA + LMWH group was lower than that in the LDA only group.
Pregnolato F et al. [25]	2021	Retrospective Single-Center	Includes the following: (1) Criteria aPL group (2) Low-titer aPL group (3) Control group (patients with SAD and negative aPL) No inclusion of non-classical aPL	Criteria aPL: 100 Low-titer aPL: 55 Control: 226	- To evaluate the impact of aPL positivity fulfilling classification criteria (‘criteria aPL’) and at titers lower than thresholds considered by classification criteria (‘low-titer aPLs’)	- An association between every single aPL test and also low-titer aPLs and pregnancy morbidity. - LA and aβ2GPIs IgG are the strongest predictors of pregnancy morbidity. - Women with low-titer aPLs benefited from SoC treatment and its effectiveness was greater than for criteria aPLs.
Alijotas-Reig et al. [7]	2020	Partially Retrospective Multicentric (30)	Includes the following: (1) OAPS group (2) NC-OAPS group (aPL-related pregnancy morbidity, low-titer and/or non-persistent aPL) Includes patients with SAD No inclusion of isolated non-classical aPL positivity	OAPS: 1000 NC-OAPS: 640	- To compare features and fetal–maternal outcomes between women with OAPS and NC-OAPS	- Similar percentages of treatment in the OAPS (77%) and NC-OAPS groups (76.09%). - Similar fetal–maternal outcomes in both groups after SoC treatment: live birth rate of 85% in OAPS and 89.6% in NC-OAPS.
Xi F et al. [47]	2020	Prospective Single-Center	Includes the following: (1) APS group (2) NC-APS group (not defined) (3) Control group (healthy pregnant women) Includes patients with SAD No inclusion of non-classical aPL	APS: 44 NC-APS: 91 Control: 135	- To investigate pregnancy outcomes of women with aPL positivity - To assess risk factors associated with APOs	- The live birth rate was 95.5% in APS and 97.8% in NC-APS. - The total use of LMWH in the APS group was significantly more than in NC-APS and the number of patients who took HCQ in the APS group was significantly lower than in NC-APS. - After treatment, the incidence of IUGR was higher in the APS group than in the NC-APS group, and both were higher than in the control group.
Ofer-Shiber S et al. [48]	2015	Retrospective Single-Center	Includes the following: (1) APS: criteria aPLs with vascular thrombosis and/or pregnancy morbidity (2) NC-APS: low-titer aPLs with vascular thrombosis and/or pregnancy morbidity	APS: 126 NC-APS: 117	- To determine the clinical manifestations and outcome of patients with persistently low (20–40 U) aCLs or aβ2GPIs IgG/IgM titers compared with those with persistent moderate–high titers and/or positive LA	- The low titer of ACL/aβ2GPIs IgG/IgM was significantly associated with an increased risk of thrombotic and obstetrical manifestations of APS similar to the risk found in patients with moderate-to-high titer.
Mekinian A et al. [21]	2012	Retrospective Single-Center	Includes the following: (1) APS (2) NC-APS (low-titer aPLs) (3) Control (seronegative APS group) Excludes other SADs No inclusion of non-classical aPLs or LAs	APS: 25 NC-APS: 32 Control: 21	- To assess whether the presence of low-titer aPL might be associated with APS-like obstetrical events - To analyze the impact of treatment with LDA and/or LMWH in patients with low-titer aPL levels	- Pregnancy outcomes in untreated patients with NC-APS are poor and similar to those in obstetrical patients with confirmed APS. - Conventional APS treatment substantially improved pregnancy and neonatal outcomes in both groups of patients. - The total number of obstetrical events per patient decreased significantly in both groups after treatment to reach an identical median value (from 3 [1–8] to 0 [0–2] in group 1 (*p* < 0.05) and from 3 [1–6] to 0 [0–2] in group 2 [*p* < 0.05]).

Abbreviations: aβ2GPIs: anti-β2 glycoprotein-I antibodies; aCLs: anticardiolipin antibodies; aPLs: antiphospholipid antibodies; APOs: adverse pregnancy outcome; APS: antiphospholipid syndrome; LA: lupus anticoagulant; LDA: low-dose aspirin; LMWH: low-molecular-weight heparin; NC-OAPS: non-criteria antiphospholipid syndrome; OAPS: obstetric antiphospholipid syndrome; SADs: systemic autoimmune diseases; SoC: standard of care; IUGR: intrauterine growth restriction.

### 7.3. Personalized Approaches and Future Perspectives

There are some studies that approach the management of one particular subset of NC-OAPS. The EUREKA algorithm was specifically designed to stratify the probability of obstetric complications in patients with different aPL titers (medium–high titers, as required for the classification criteria for APS, and low titers, lower than the threshold considered by the classification criteria) and the effectiveness of the therapy based on the aPL profile [25]. All women with low-titer aPLs benefited from treatment with LDA+LMWH±HCQ, and its effectiveness was even greater for low-titer than for criteria aPLs [25]. Other studies also reported that the appropriate treatment of patients with low aPL levels led to better pregnancy outcomes (Table 3) [21,45]. Some studies found that patients with aPS/PT also had a reduction in pregnancy losses if they received treatment during their pregnancy [30,49,50]. Furthermore, recent retrospective studies found that treatment with LDA plus LMWH +/− HCQ improved IVF outcomes in patients with aPL, increasing the clinical pregnancy rate, implantation rate, and take-home baby rate [51,52].

Apart from LDA and LMWH, no other treatments have robust evidence of effectiveness in OAPS or NC-OAPS. Experimental studies have shown that HCQ can prevent the negative effects of aPLs in the placenta through anti-thrombotic, anti-inflammatory and immunomodulatory mechanisms (reviewed by Andreoli L et al.) [53]. Retrospective studies of pregnant women with OAPS or aPL carriers also suggested a beneficial effect of HCQ in addition to conventional treatment, with negligeable side effects [54,55]. Based on these data, HCQ is recommended as an add-on therapy in refractory OAPS and, by extrapolation, may be considered in refractory NC-OAPS [36]. The HYPATIA trial is an ongoing prospective randomized controlled trial, currently recruiting, aiming to find out if HCQ improves pregnancy outcomes in women with aPLs [56]. Evidence favoring the use of corticoids in the setting of OAPS and NC-OAPS is less compelling. Glucocorticoids did not appear to improve pregnancy outcomes in early trials. However, recent studies in women with refractory APS-associated pregnancy loss and the use of lower glucocorticoid doses have re-established interest in this therapy. A meta-analysis of these studies showed that glucocorticoids, in combination with anti-thrombotic treatment, tended to increase the frequency of a successful pregnancy, although this effect was not statistically significant (OR = 0.509, 95% CI 0.252–1.028, *p* = 0.06). It also found that glucocorticoids increased the frequency of PE and gestational diabetes, particularly in studies using higher glucocorticoid doses. Due to the high heterogeneity of the studies included in the meta-analysis, in terms of the dose and duration of corticosteroid treatment, it was not possible to draw clear conclusions, but the authors open the possibility that low-dose prednisone (≤10 mg/day), particularly when discontinued around 12 weeks of gestation, may have a place in this setting, although more studies are needed [57]. The lack of a solid benefit together with the unfavorable safety profile of corticosteroids during pregnancy turn them a last resource option. In our personal clinical practice, we use HCQ the same way as in OAPS, in refractory cases, starting this treatment pre-conceptionally. We reserve the use of corticosteroids to refractory recurrent miscarriages and refractory RIF, in low-doses and until 12 gestational weeks, with a subsequent reduction over 4 weeks. Intravenous immunoglobulins (IVIGs) have also been explored as a treatment option. A meta-analysis showed varying levels of success in improving live birth rates and reducing miscarriage rates through IVIG intervention in aPL-positive patients with recurrent miscarriage, with this effect being more prominent and statistically significant in aPL-positive patients in combination with systemic lupus erythematosus or other similar autoimmune diseases, but the IVIG-treated group exhibited a higher incidence of preterm labor [58]. In a posterior multicenter clinical trial, an IVIG-only add-on intervention for refractory OAPS did not demonstrate efficacy in improving the proportion of live births after 30 weeks of gestation [59].

Given the limited and low-quality data concerning the treatment of patients with NC-OAPS, no formal recommendations exist. Faced with this conundrum, physicians should personalize treatment according to an individual’s risk profile and a discussion with the woman, weighting the potential risks and estimated benefit of treatments in each scenario. Known risk factors for adverse events include a poor reproductive history (a history of ≥ four pregnancy losses), a high-risk antibody profile (a higher number of positive aPLs such as triple or double positivity or high aPL titers), concomitant systemic lupus erythematosus and/or other autoimmune diseases, and hypocomplementemia (which appears to be a promising tool to predict APOs in the context of OAPS) [60]. In this equation, physicians may also consider additional risk factors for pregnancy loss or thrombosis, advanced maternal age, or IVF pregnancy [39]. It should be noted that LMHW is considered a safe treatment during pregnancy. A low risk of significant bleeding (1.98%, generally associated with primary obstetric causes), low percentages of allergic skin reactions (1.80%) and osteoporotic fractures (0.04%), and no cases of heparin-induced thrombocytopenia were documented in a systematic review of the studies addressing the safety of LMWH use in pregnancy for several indications (the treatment of venous thromboembolism or thromboprophylaxis or the prevention of APOs) [61]. The adverse effects of the combination of LMWH and aspirin are not consistently reported in studies, with uncertainty as to how this association impacts the bleeding risk [37]. Nevertheless, a multicenter study evaluating the safety of antithrombotic treatments during pregnancy in patients with APS, the majority of which were treated with the combination of LMWH and aspirin (215 in 264 pregnancies, 81%), concluded that the treatment was safe, with major bleeding reported in 3% of pregnancies, a rate similar to that described in the general population [62].

The majority of studies on OAPS focus on the treatment strategies aiming to prevent repeated APOs. However, outside the context of pregnancy, patients with pure OAPS have been shown to be at risk of future thrombosis, with a wide range of cumulative risk or incidence rates described in the different articles (from 10.4% to 63% in over 10 years and an incidence rate of 3.3–4.9/100 patient-year) [63,64,65,66]. A study also found that the risk remains higher than in patients without aPLs even under aspirin treatment [67]. The several studies pointed out diverse predictors of future thrombosis events, such as multiple aPL positivity, the presence of antinuclear antibodies, heart valve disease, a younger age of OAPS, higher adjusted Global Antiphospholipid Syndrome score (aGAPPS) and systemic lupus erythematosus [63,64,66]. The applicability of these observations to long-term outcomes in women with NC-OAPS has not been determined, and there is no agreement regarding the benefit of prescribing prophylactic anti-thrombotic treatment to these women.

## 8. Discussion

NC-OAPS is a heterogenous condition that comprises both diverse clinical manifestations and aPL profiles. This characteristic, per se, complicates its investigation, as published studies have very diverse study populations and designs. In this review, we tried to describe the available evidence focusing on each NC-OAPS feature, remarking that the different scenarios are associated with different grades of evidence and diverse clinical impact.

Currently, evidence from observational studies, mostly retrospective, tends to suggest that patients with NC-OAPS are at a risk of APOs and may benefit from treatment. Given the lack of high-quality evidence, there are no formal recommendations on the management of these patients. Regardless, many patients with NC-OAPS are treated similarly to those with OAPS [40]. One possible explanation for this fact is that clinicians tend to offer a safe treatment and patients are eager to take it when faced with APOs in the context of aPL presence, although accepting a possible and unknown proportion of overtreatment. Unfortunately, because of the heterogenous phenotypes of NC-OAPS and the incompletely known impact of each subtype, it would be imprudent to make a generalized recommendation for the management of NC-OAPS. For example, is a patient with two early pregnancy losses and persistent double aPL positivity in low titers at the same risk of a future APO than a patient with one fetal death because of severe PE only carrying aPS/PT antibodies? Is treatment equally beneficial in these two situations? In light of the current scientific knowledge, these questions do not have a definite answer.

It is worth remembering that before considering the diagnosis of NC-OAPS and offering treatment to women with APOs and transitory or dubious aPL titers, the other (and more common) causes of APOs should not be overlooked, and a comprehensive investigation should be performed and managed accordingly. In the case of recurrent pregnancy losses, it is recommended to make an anatomical evaluation (for congenital and acquired uterine malformations), thyroid function assessment, parental karyotyping, and genetic analysis of pregnancy tissue, as well as lifestyle counseling (to avoid smoking, alcohol, obesity or underweight) [68,69]. Multiple and sometimes overlapping insults can also be responsible for PE, such as gestational diabetes mellitus, metabolic syndrome, pre-existing kidney disease, autoimmunity diseases (particularly, systemic lupus erythematosus), endocrine disorders and maternal infections. These must be acknowledged when considering the role of aPLs in this condition.

Future studies appropriately designed and with adequate power should analyze the different subsets of NC-OAPS with well-defined and homogenous NC-OAPS populations, in order to clarify discrepancies and similarities among them and suggest management specificities. Especially in what refers to treatment in the different scenarios of NC-OAPS, randomized controlled trials should be conducted before a treatment is generalized. However, these trials are recognizably difficult to achieve, as was shown by the pilot APPLE (AntiPhospholipid syndrome low-molecular-weight heparin Pregnancy Loss Evaluation) trial. This trial was designed to evaluate LMWH/aspirin versus aspirin prophylaxis alone during pregnancy in women with a history of ≥two early pregnancy losses or ≥one late pregnancy loss and positive aPLs according to the Sydney criteria. It was not possible to conclude the recruitment of the study because many patients wanted to use LMWH/aspirin during pregnancy in the hope of improving pregnancy outcomes [70]. Furthermore, although APS classification criteria were created for the inclusion of patients in trials, these should not preclude the conduction of high-quality studies including women not fulfilling these criteria.

Another line of research urgently needed is the impact of NC-OAPS, in its different versions, on long-term outcomes and its best management. This is an understudied topic, which will become increasingly relevant as more and more women are getting tested for aPLs and getting treated during pregnancy.

## 9. Conclusions

While NC-OAPS is a heterogeneous condition and ambiguously described in the literature, it is an increasingly recognized reality among women facing difficulties with their pregnancies. Presently, evidence suggests that it may have a significant although variable impact on pregnancy outcomes. There are, however, many current gaps in the knowledge on this topic that deserve an appropriate and high-quality investigation effort. We propose a crucial although ambitious research agenda: (1) a multicenter randomized controlled trial, with clearly defined inclusion/exclusion criteria, focusing on the impact of each particular subgroup of NC-OAPS and the benefit and risk of treatment strategies; and (2) a properly conducted long-term follow-up study aiming to explore the long-term impact of NC-OAPS on women’s health and its ideal preventive strategies.

## Figures and Tables

**Table 1:** The 2023 ACR/EULAR OAPS classification criteria.

Clinical Domains	Weight
>3 Consecutive pre-fetal (<10 w) and/or early fetal (10 w–15 w 6 d) deaths	1
Fetal death (16 w–33 w 6 d) in the absence of PE with severe features or PI with severe features	1
PE with severe features < 34 w or PI with severe features with/without fetal death	3
PE with severe features < 34 w and PI with severe features < 34 w with/without fetal death	4
**Laboratory domains**	
One-time positive LA	1
Persistent positive LA	5
Moderate or high positive IgM (aCL and/or aβ2GPI)	1
Moderate positive IgG (aCL and/or aβ2GPI)	4
High positive IgG (aCL or aβ2GPI)	5
High positive IgG (aCL and aβ2GPI)	7

At least 3 points from clinical domains plus at least 3 points from laboratory domains is required to classify as OAPS. Abbreviations: aβ2GPI: anti-β2 glycoprotein-I antibody; aCL: anticardiolipin antibody; LA: lupus anticoagulant; OAPS: obstetric antiphospholipid syndrome; PE: pre-eclampsia; PI: placental insufficiency; w: weeks.

**Table 2:** Proposed “non-criteria” OAPS subsets.

	Seronegative OAPS	Clinical Non-Criteria OAPS	Incomplete Laboratory OAPS	Laboratory Non-Criteria OAPS
**Clinical features**	Criteria + non-criteria manifestations	Non-criteria manifestations	Criteria manifestations	Criteria manifestations
**aPL profile**	Persistently negative aPLs	aPL positivity fulfilling the APS laboratory criteria	Persistently positive but low-titer criteria aPLs	Persistently negative or low-titer criteria aPLs, but positive aPLs different from LA/aCLs/aβ2GPIs IgM or IgG isotypes

Abbreviations: aβ2GPIs: anti-β2 glycoprotein-I antibodies; aCLs: anticardiolipin antibodies; aPLs: antiphospholipid antibodies; APS: antiphospholipid syndrome; LA: lupus anticoagulant; OAPS: obstetric antiphospholipid syndrome.
